# Bonus-based mercenary punishment promotes cooperation in public goods games

**DOI:** 10.1016/j.heliyon.2023.e22748

**Published:** 2023-11-29

**Authors:** Hongwei Kang, Shaoxiang Liu, Qingyi Chen, Yong Shen, Xingping Sun

**Affiliations:** School of Software, Yunnan University, Kunming 650000, China

**Keywords:** Cooperation, Bonus, Mercenary punish, Subsidy, Public goods game

## Abstract

Various regions often adopt punish strategies to solve traffic congestion problems. Punishing defectors is an effective strategy to solve the first-order free-rider problem in a public goods game. But this behavior is costly because the punisher is often also involved in the original joint venture and therefore vulnerable, which jeopardizes the effectiveness of this incentive. As an option, we could hire special players whose sole duty would be to monitor the population and punish defectors. The fines collected by various regions will also be used to subsidize the construction of public transportation. Thereby, we derive inspiration, and propose an improved public goods game model based on bonus and mercenary punishment. Research has shown that after cooperator gives the punisher an appropriate bonus, cooperators can strengthen the punisher, thereby weakening the defector's advantage and indirectly promoting cooperation by stabilizing the punisher's position in the system. In addition, the mechanism of reusing the fines collected from defectors and then subsidize to other players in the system can directly promote the emergence of cooperation.

## 1 Introduction

Darwin's theory of evolution [1], emphasizes selfishness is an individual's instinct. Rational individuals often choose to defect, leading to the tragedy of the commons. However, cooperative behavior is a common phenomenon in biological systems and human societies [[2], [3], [4], [5]]. Therefore, how to understand cooperative behavior in selfish groups [6,7] and promote cooperation in the system has become the focus of many scholars' research. Game theory has led to the proposal of classic game models, such as prisoner's dilemma game [8], snowdrift game [9], deer hunting game in the two-person game model, and the public goods game in the multi-person game model [[10], [11], [12]]. The public goods game is also known as the multiplayer prisoner's dilemma game. This game includes N participants, each of whom has the freedom to choose whether to invest in a public goods pool, with the accumulated investment amount increasing by R times (1< r < N) and is evenly distributed to each participant(Regardless of the participant's choice). From the perspective of personal rationality, the best choice for an individual would be to defect. It can be imagined that if this game continues, no one will ultimately choose cooperate. However, from the perspective of collective rationality, only when everyone contributes can the entire group receive the highest return, referred to as the “tragedy of the commons.”

To explain social dilemmas, Nowak summarized five typical mechanisms for promoting cooperation based on his previous work: kinship selection used to explaining altruistic behavior in organisms; direct reciprocity of mutual exchange, reciprocity and mutual benefit between among individuals; indirect reciprocity for establishing indirect reciprocal relationships through the construction of reputation systems; and network reciprocity [[13], [14], [15], [16], [17]]; and group selection for exploring the influence of network structure on the spread and diffusion of reciprocal behavior. Among them, network reciprocity based on structured populations [[18], [19], [20], [21], [22]] has gained a strong influence in this field. To improve the level of cooperation in the system, many scholars have proposed relevant mechanisms, such as, reputation [[23], [24], [25]], rewards and punishments [[26], [27], [28], [29]], expectations [[30], [31], [32]], social diversity [[33], [34], [35]], investment heterogeneity [[36], [37], [38], [39], [40]], and pool exclusion [[41], [42], [43], [44]], all of which can promote the evolution of cooperation. The punishment mechanism has aroused our interest. The analysis of pure punishment and pure reward mechanisms [27] provides valuable insights on how to design appropriate mechanisms to promote cooperation and reduce selfish behavior. We have decided to continue our research in this area.

To address tragedy of the commons, some scholars have proposed punishment strategy, that is, the punisher paying a certain price to punish the defector, making the defector pay a fine, thus weakening the defector's advantages. This paper draws inspiration from the actions of governments worldwide in solving traffic congestion. To resolve the congestion problem, many governments adopt similar punishment strategies, which increase the cost of owning private cars, including increasing parking fees in urban areas and continuously increasing the purchase price of private car licenses. However, governments not only take punitive measures, but also use the fines collected for public transportation projects, such as building subways and expanding bus routes. Once the defector pays the fine, the fund should be utilized and subsidize to other players in the system. In the standard public goods game, the defector can divide the funds in the fund pool without investing anything. We think this is a first-order free ride. In our model, we adopted the mercenary punishment model [45], but a pure mercenary punishment is a pure punisher system that leads to a state of complete betrayal [46]. Therefore, we introduced a bonus mechanism whereby collaborators reward the hired punisher to strengthen the punisher, thereby weakening the advantage of the defector and indirectly promoting cooperation by stabilizing the punisher's position in the system.

## 2 Model

According to the rules of the public goods game, each player will participate in a total of five games centered around themselves and their neighbors in each round. In this game, if a player chooses a cooperation strategy, they not only have to pay a certain amount of cooperation cost(c), but also pay a bonus(b) to maintain the punisher's position in the system. In addition, the cooperator will receive a portion of the defector's fine(fine represents the penalty paid by the defector as punishment by the punisher). If the player chooses a defection strategy, they do not need to pay the cost of cooperation, but as long as there are punishers in the group, the defector must pay a certain fine. However, if there are no punishers in the group, the group will automatically transform into a standard public goods game. Alternatively, if the player chooses a punishment strategy, they do not need to pay the cost of cooperation, but need to pay a certain penalty cost(x) when punishing the defector. The fines paid by the defector will be subsidied to other players in the system, some to the cooperators(α) and the other to the punishers(β). This will be used to study who the defector 's fines are subsidied back to, which will have a more significant promoting effect on cooperation. The profits of players with different strategies in the group in the game can be expressed as follows:

(1) $$\pi_{i}=\left\{ \;\begin{array}{c} \frac{rcN_{c}}{N}-c-b+\frac{\alpha N_{d}fine}{N_{c}},if\;S_{i}=C\;\\ \frac{rcN_{c}}{N}-fine,if\;S_{i}=D\;\\ \frac{rcN_{c}}{N}+\frac{bN_{c\;}+\beta N_{d}fine\;}{N_{p}}-x,if\;S_{i}=P\; \end{array}\right.$$

This paper uses Monte Carlo simulation to update the player's strategy in the system. Evolutionary games update strategies based on given random imitation rules and iterate forward. After each game round, each player i randomly selects one of their neighbor j, and the probability of adopting the neighbor j’s strategy is as follows:

(2) $$\begin{array}{c} W\left(S_{i}\;\leftarrow \;S_{j}\right)=\frac{1}{1+\exp\left[\frac{\pi_{i}-\;\pi_{j}}{K}\right]}, \end{array}$$

where K represents the uncertainty or so-called random noise caused by strategy adoption. In our research, the noise K is set to 0.1(K represents the uncertainty of the strategy, meaning that player strategies with higher returns are more likely to be adopted, but there is still a certain probability of adopting player strategies with lower returns). This parameter quantifies the uncertainty of strategy adoption, meaning that players with higher returns are more likely to adopt strategies, but there is also a possibility of adopting strategies with lower returns.

## 3 Simulation results

In this paper, three strategies have the same proportion in the initial stage and all experimental data are obtained after at least $10^{7}$ iterations. To avoid randomness in the experiment, all experimental data are averaged after ten repeated experiments.

**Fig. 1:**
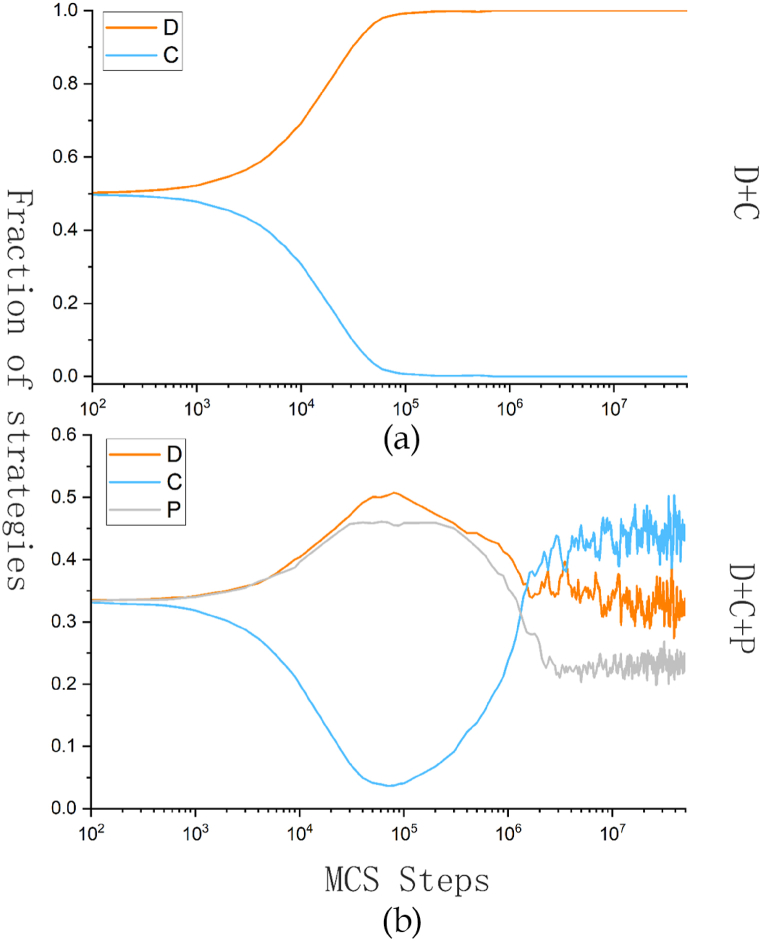
The evolution of the proportion of each strategy in different strategy combinations over time.

Next, the impact of penalty strategies on game evolution is investigated. Fig. 1 compares the snapshot of the changes in the proportion of each strategy before and after the introduction of penalty strategies. In Fig. 1, the main parameters are set as follows: r = 3.5, fine = 0.2, T = 0.05, cost = 0.32, and α = β = 0.5. We conducted preliminary research on the model. This paper introduces a mercenary punishment strategy based on bonus support in the standard public goods game and compares the evolution results of two different strategy combinations. In the standard public goods game shown in Fig. 1(a), the profits of cooperators are lower than those of defectors, making it impossible to resist their invasion. Therefore, the proportion of cooperators rapidly decreases, ultimately leading to the extinction of all cooperators. After introducing the punishment strategies based on bonus support in Fig. 1(b), the situation significantly changes compared with before, and the level of cooperation greatly improves compared with that in the standard public goods game. Therefore, we can see that this mechanism is effective in preliminary research. The reason for this phenomenon is that in the C + D model, the cooperators do not have the ability to resist betrayal. When the synergy factor r is not large enough, cooperators’ benefits are much lower than that of defectors, so the cooperators are quickly invaded by the defectors until they perish. In the C + D + P model, due to the existence of bonus supported punishers to assist cooperators in resisting defectors, the cooperation strategy can survive and ultimately evolve into a stable system where three strategies coexist and cooperation dominates.

**Fig. 2:**
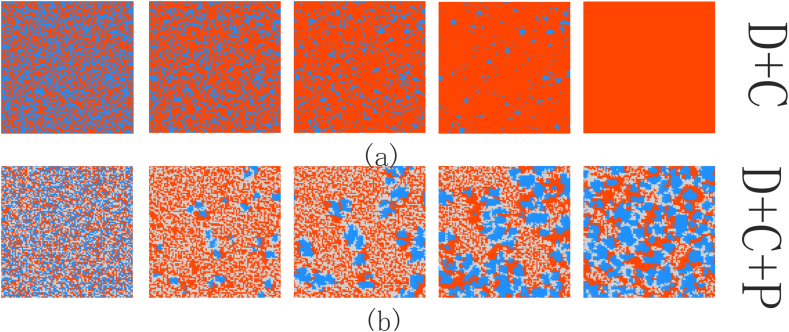
Snapshot of the game evolution under different strategy combinations.

Fig. 2(a) and (b) show the evolution snapshots of (C + D) and (C + D + P), respectively.Each time step from left to right is 10^0, 10^4, 10^5, 10^6, and 10^7. Among them, red represents defectors, blue represents cooperators, and gray represents punishers. The same color scheme applies to other content in this paper.

In the standard public goods game (C + D), early defectors quickly invade cooperators, and cooperators quickly decrease and gradually gather to form many smaller clusters. However, at this point, the network reciprocity between the cooperators clusters is not enough to resist the invasion of the defectors, leading to the eventual extinction of cooperators. In a game composed of three strategies, various strategies are randomly scattered throughout the entire grid in the initial state, making it very conducive to the exploitation of cooperators by defectors. Therefore, in the early stages, the number of cooperators quickly decreases, while the number of defectors and punishers quickly increases. In the subsequent evolution process, cooperators gradually form clusters, and punisher surround them. This allows the punishers to continuously punish the defectors, neutralizing the defector's advantage and gradually increasing benefits for cooperators after forming a cluster. The final three strategies form interlocking clusters, and they dynamically maintain balance. Thus, it can be observed that the model has a good enhancing effect on cooperation under the same gain factor (r = 3.5).

**Fig. 3:**
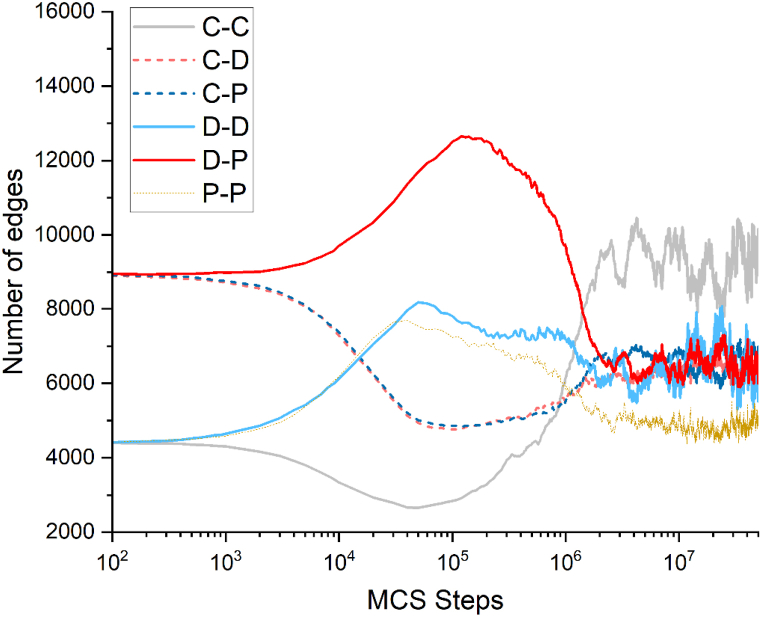
The result of the evolution of the number of various types of edges in the system over time.

During the evolution, the initial number of cooperators rapidly decreases and they converge into clusters in Fig. 2. To fully explain the phenomenon presented in Fig. 2, we study the dynamic evolution of the game from the perspective of the number of connected edges, as shown in Fig. 3. In Fig. 3, the edges (C–C, C-D, C–P) related to cooperators decrease, while the number of defectors and punishers continues to increase. Thus, the number of edges unrelated to cooperators (D-D, D-P, P–P) continues to increase. When D-P consecutive edge reaches a high level, which means that a large number of defectors will be punished, at this time, the defectors do not have an advantage in terms of the gains relative to the cooperators, and the defectors may then learn the cooperative strategy or the punishment strategy. In the subsequent evolution process, the clusters of cooperators gradually expand, and punishers surround them. Therefore, at this stage, the connections between C–C and C–P gradually begin to increase. The continuous punishment of defectors by the punishers leads to a sharp decrease in the number of defectors. After the number of defectors reaches its peak and begins to decline, the edges (D-D, D-P, D-C) related to the defection strategy begin to decrease. Finally, the three strategies reach a dynamic equilibrium, and the number of each type of edge connection tends to change dynamically.

**Fig. 4:**
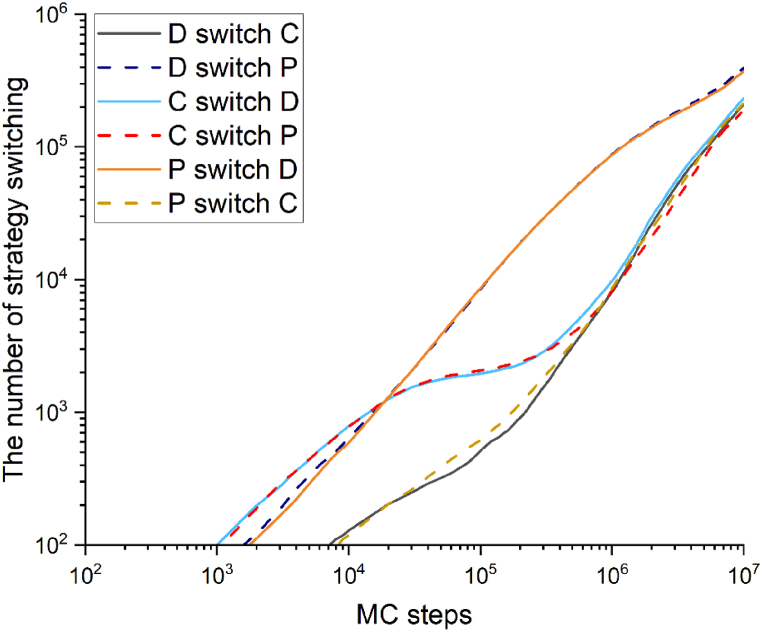
The result of the evolution of the number of policy transitions in the system over time.

Fig. 4 shows the mutual transformation between various strategies throughout the entire evolution process. The first curve to appear and grow represents the transition from cooperative strategies to other strategies (C → D, C → P) because the number of cooperators is rapidly decreasing at this time. Subsequently, these two curves enter a stable stage, as the cooperators form clusters and continue to expand, giving them an advantage in the entire system. Correspondingly, the curves that depict the conversion of other strategies into cooperative strategies continue to grow (D → C, P → C).

**Fig. 5:**
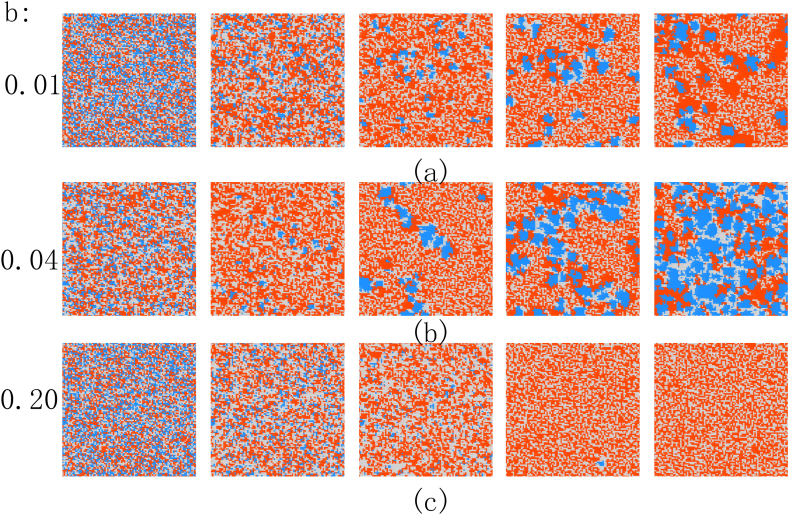
Snapshot of the policy distribution under different b conditions when r = 3.5, fine = 0.2, and c = 0.32 are fixed.

In order to investigate the role of network reciprocity in our model, we drew a snapshot of Fig. 5 using the variation of b parameters as an example. Fig. 5 reveals that, the relationship between the bonus coefficient b and the level of cooperation is not a simple positive correlation, but rather denotes an appropriate range of bonus coefficient b values that can lead to the optimal level of cooperation. When the bonus coefficient b in Fig. 5(a) is 0.01, the value of the bonus coefficient b is too small to provide sufficient support to the punisher, giving the defector an advantage. When the bonus coefficient b in Fig. 5(c) is 0.2, the heavy burden on the cooperators places them at an absolute disadvantage within the entire system, leading to their quick elimination. Without cooperators, the overall system's revenue is basically zero, and ultimately the entire grid is left only with defectors and punishers. When the bonus coefficient b in Fig. 5(b) is moderate, the cooperators not only provide sufficient support for the punisher, but also ensure that their own burden is not too heavy. This balance allows the punisher to continuously weaken the advantages of the defector and, compress the Lebensraum of the defector, while the cooperator survives under the protection of the punisher, and the defector chooses to stick close to the cooperator. Therefore, the punisher, cooperator and defector form an interlocking cluster.

In our model, network reciprocity exerts great impact on the emery of cooperation. The reason for the first decrease and then increase in the number of collaborators in Fig. 1 is also reflected in Fig. 5 only when cooperators gather together can they improve their mutual benefit through network reciprocity, thereby enhancing the competitiveness of the cooperator community to resist the invasion of defectors. This is because neither the collaborator nor the punisher can survive alone without the other party. The collaborator needs the shelter of the punisher, and the punisher also needs the compensation of the collaborator. At the beginning of evolution, such a mutually beneficial and symbiotic network structure was clearly not formed, and at this time, collaborators were heavily invaded by defectors. As collaborators gather, punishers begin to appear around them, and the two work together to invade the defector. In the fine feedback system, collaborators can receive more fines than the punisher, which establishes their competitive advantage. Consequently, the number of cooperators becomes the majority among the players in the system, and they coexist with defectors and punishers.

**Fig. 6:**
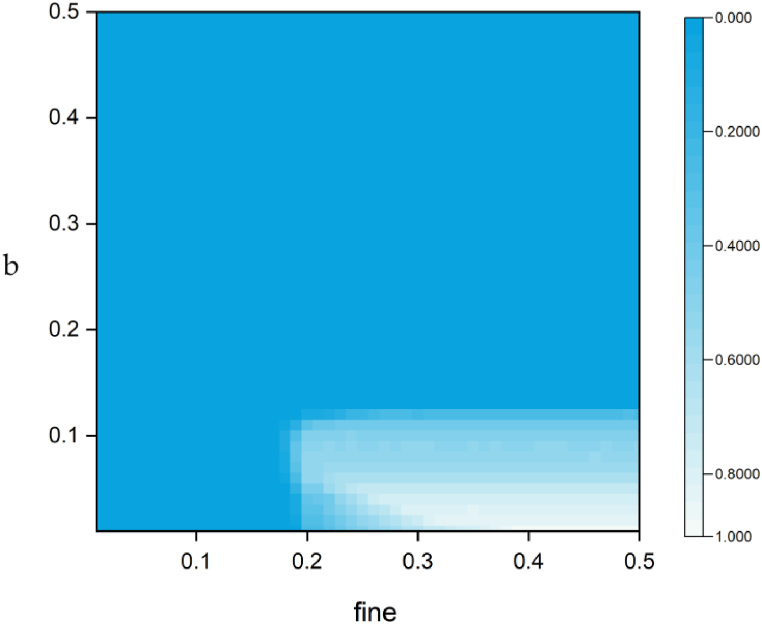
The proportion of cooperative strategies as a function of (b, fine).

Subsequently, we conducted research on cooperation in different parameter spaces and drew heat maps. In Fig. 6, when the fine is greater than 0.3, the punishment for the defector becomes too strong, resulting in the punisher clearing the defector from the system. Consequently, only cooperators and punishers remain, and the parameter b shows a negative correlation with the cooperator level. Therefore, this paper focuses on the influence of parameters when three strategies coexist. For example, when b is between 0.2 and 0.3, the cooperation level becomes a non-monotonic function of the bonus coefficient b, which indicates that an appropriate b interval can lead to the optimal cooperation level. When b initially increases, although it increases the burden on some cooperators, all bonus are ultimately handed over to the punisher. Consequently, punishing the defector by increasing the punisher's support can also achieve the effect of improving the level of cooperation. When b becomes too large, the burden on the cooperators becomes too heavy, and they will eventually be eliminated from the entire system.

**Fig. 7:**
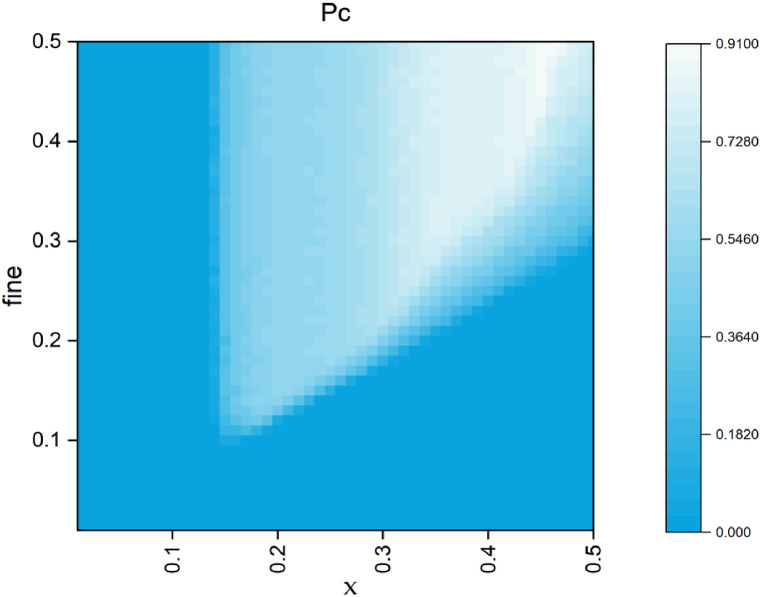
The proportion of cooperative strategies as a function of (fine, x).

In Fig. 7, when the fine coefficient is small, the punishment for defectors is insufficient, and cooperation cannot emerge. The larger the fine coefficient, the greater the punishment for the defector, and this fine can also be subsidied back to other players in the system, continuously improving the level of cooperation. The continuous increase in the fine coefficient will suppress the spread of defectors.Simultaneously, the increase in the fine coefficient increases the severity of punishment for the defector, further weakening the advantage of the defector population. Moreover, as the fine coefficient increases, the strength of other players in the fine feedback system becomes greater. In the process of group selection, the advantage of the cooperator population exceeds that of the defector population, and the cooperator population wins the competition. However, for the cost of punishment, it cannot be blindly increased, as it will lead to the punisher losing their competitive advantage and thus unable to effectively suppress the growth of defectors. So only by increasing the cost while also obtaining more compensation funds through increasing the penalty coefficient can the cooperative group survive.

**Fig. 8:**
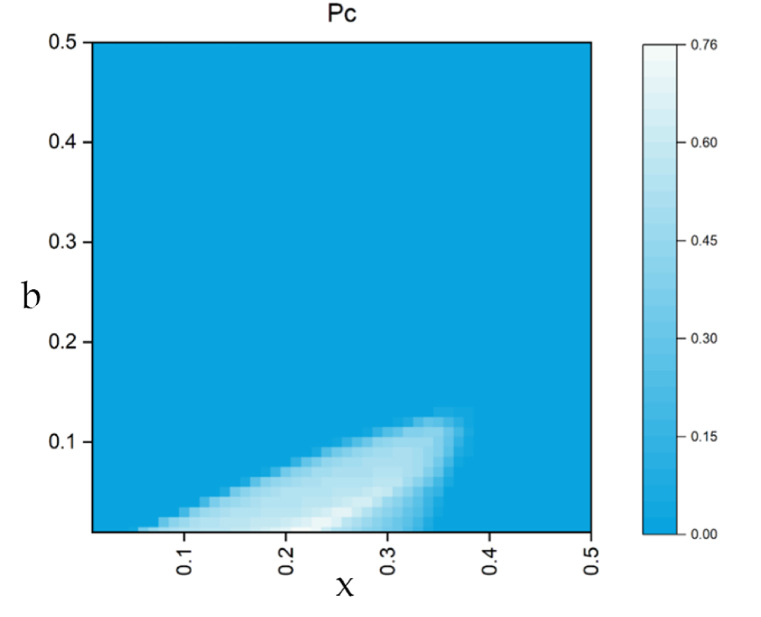
The function between the proportion of cooperative strategies and (b, x).

In Fig. 8, it is evident that when the cost is at both low and high values, the cooperators cannot survive, but the mechanism behind this phenomenon differs. At the beginning, with a small increase in bonus b, the cost of the punisher also needs to increase, in order to ensure that collaborators have a competitive advantage over the punisher, while also suppressing the breeding of defectors. However, if the cost is too high, the punisher will be quickly encroached upon, and collaborators who lose the protection of the punisher will naturally not survive, and the system will be dominated by defectors. Similarly, excessive bonus is also a burden for collaborators, which weakens the impact of punishers on them.

**Fig. 9:**
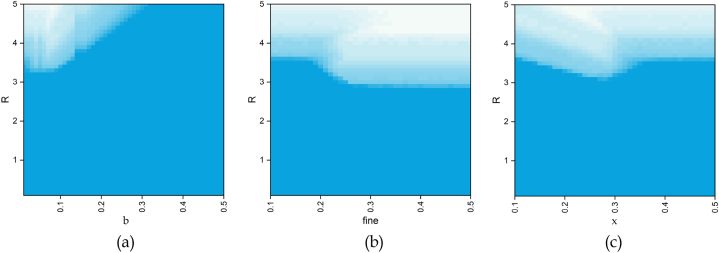
The function of the proportion of cooperators between the gain factor R and other parameters.

In Fig. 9, a gradient from blue to white represents an increase in the cooperation ratio from 0 to 1. We can observe that in the standard public goods game, cooperation needs to emerge when R is greater than 3.5. However, after introducing the bonus support punishment mechanism, the threshold for cooperation to emerge decreases. For the bonus coefficient b in Fig. 9(a), an excessively large b value will make the burden on the cooperators too heavy, and a small value of b cannot support the punisher well. For the fine coefficient in Fig. 9(b), an increase in the value of the coefficient will weaken the advantage of the defector and thus enhance the level of cooperation. For the punishment cost coefficient x in Fig. 9(c), a smaller cost will give the punisher a dominant position, while a larger cost coefficient will result in the punisher no longer having an advantage over the defector and no longer being able to suppress the defector.

**Fig. 10:**
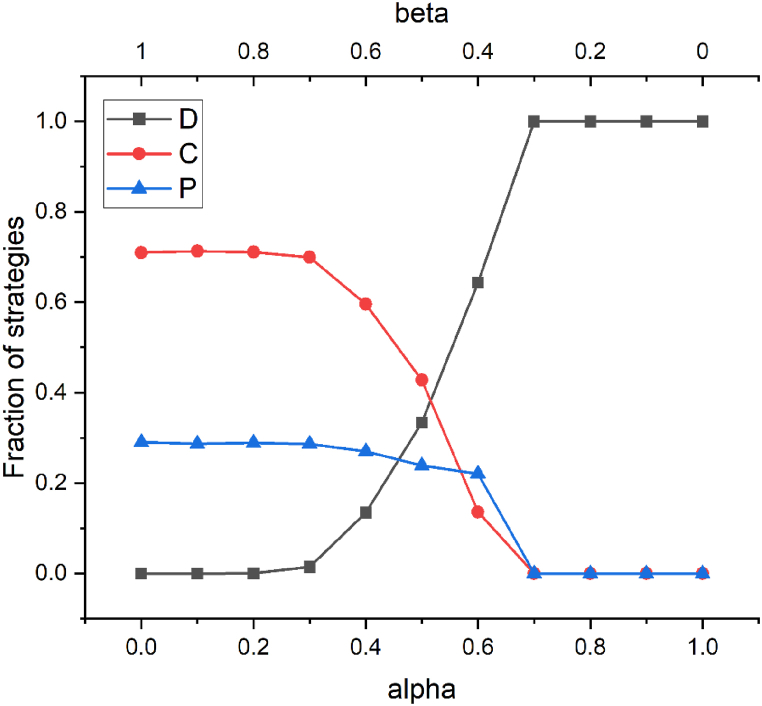
When r = 3.5, b = 0.05, fine = 0.21, x = 0.32, and for different α and βvalues, the function between the parameters and the proportion of each strategy (α+β = 1) is shown.

In this scenario, the fines paid by the defector are collected and continue to be subsidied back to other players in the system. Previously, in this study, the fines were divided equally between the cooperators and punishers. In Fig. 10, we study how the collected fines can be allocated to better promote the emergence of cooperation. When the parameter α increases, which means that the allocation of fines tends to be more towards the cooperators, the level of cooperation in the system will be lower. This is because by allocating more fines to cooperators, the rewards of the punisher will decrease. Consequently, the punisher is weakened and cannot play the role of punishing the defector. As a result, it is only a matter of time before the cooperator is eliminated by the defector once the punisher disappears. Therefore, it can be concluded that in the distribution of fines, more emphasis should be placed on the punisher. Only by ensuring the status of the punisher can the invasion of the defector be effectively restricted, thereby indirectly improving the level of cooperation.

## 4 Conclusion and discussion

This article studies the evolution of cooperative behavior with a bonus-based mercenary punishment mechanism. To maintain their benefits, cooperators are willing to hire mercenaries to punish defectors for their treachery. This research has found that there is an appropriate range for the bonus coefficient b, which allows for both the burden on the cooperators to be within an acceptable range and the strength of the punisher to be enhanced. This weakens the advantage of the defector by stabilizing the punisher's position in the system, indirectly promoting cooperation and enhancing the level of cooperation in the system. In addition, drawing on the utilization of fines in the real world, where fines are collected and continue to be subsidied back to other players in the system. Compared with the situation where cooperators pay bonus to support mercenaries, this scenario indirectly promotes cooperation, and the reuse of fines can more directly promote the emergence of cooperation.

After introducing a bonus support mercenaries mechanism, the threshold for cooperation to emerge decreases. In the distribution of fines, greater emphasis should be placed on the punisher. By ensuring the status of the punisher, the invasion of the defector can be effectively restricted, thereby indirectly improving the level of cooperation. For the bonus coefficient b, an excessively large b value will make the burden on the cooperators too heavy, and a value of b that is small cannot support the punisher well. For the fine coefficient, an increase in the value of the coefficient will weaken the advantage of the defector and thus enhance the level of cooperation. For the cost parameter for punishment, a smaller cost will give the punisher a dominant position, while a larger cost coefficient will also result in the punisher no longer having an advantage over the defector and being able to suppress the defector.

Punishment strategies can indeed greatly promote cooperation, but research has shown that punishment may lead to retaliation and escalation of punishment actions, ultimately causing a hostile environment and ultimately undermining the goal of cooperation. In addition, the implementation of punishment may be subjectively influenced, resulting in unfair punishment and corruption. These are all worth further exploration in our future research.

## Data Availability

No data was used for the research described in the article.

## CRediT authorship contribution statement

**Hongwei Kang:** Writing – original draft, Investigation, Funding acquisition, Formal analysis, Data curation, Conceptualization. **Shaoxiang Liu:** Writing – review & editing, Writing – original draft, Software, Resources, Project administration, Methodology, Investigation, Formal analysis, Data curation, Conceptualization. **Qingyi Chen:** Writing – original draft, Software. **Yong Shen:** Visualization, Validation, Software. **Xingping Sun:** Project administration, Methodology.

## Declaration of competing interest

The authors declare that they have no known competing financial interests or personal relationships that could have appeared to influence the work reported in this paper.
